# RNA-Seq transcriptome data of the liver of common Pekin, Muscovy, mule and Hinny ducks fed ad libitum or overfed

**DOI:** 10.1016/j.dib.2021.107516

**Published:** 2021-10-27

**Authors:** Frédéric Hérault, Elisabeth Baéza, Christian Diot

**Affiliations:** a PEGASE, INRAE, Institut Agro, Saint Gilles 35590, France; b INRAE, Université de Tours, BOA, Nouzilly 37380, France

**Keywords:** Ducks, Interspecific hybrids, Liver, RNA sequencing, Overfeeding, Hepatic steatosis

## Abstract

Duck species are known to have different ability to fatty liver production in response to overfeeding and gene expression analyses can help to characterize mechanisms involved in these differences. This data article reports the sequencing of RNAs extracted from the liver of Pekin and Muscovy duck species and of their reciprocal hybrids, Mule and Hinny ducks fed *ad libitum* or overfed. Libraries were prepared by selecting polyadenylated mRNAs and RNA Sequencing (RNASeq) was performed using Illumina HiSeq2000 platform. RNASeq data presented in this article were deposited in the NCBI sequence read archive (SRA) under the accession number SRP144764 and links to these data were also indicated in the Data INRAE repository (https://doi.org/10.15454/JJZ3QQ). Transcriptome analyses of these data were published in Hérault et al. (2019) and Liu et al. (2020).

**Specifications Table**

**Table:** 

Subject	Biological sciences
Specific subject area	Omics: Transcriptomics
Type of data	Sequence data
How data were acquired	High throughput RNA sequencing Illumina HiSeq2000 platform
Data format	FASTQ files (raw data)
Parameters for data collection	4 duck genetic types: - Common Pekin duck (*Anas platyrhynchos*, Ap) - Muscovy duck (*Cairina moschata*, Cm) - Mule hybrid (Cm x Ap) - Hinny hybrid (Ap x Cm) 2 feeding status: - Fed *ad libitum* - Overfed with corn
Description of data collection	Total RNA was extracted, then polyA RNA fraction was enriched and used for construction cDNA libraries using a Illumina TruSeq RNA Sample Prep Kit v2. RNA sequencing was performed on a Illumina HiSeq2000 using a paired-end read length of 2 × 100 pb with the Illumina HiSeq2000 SBS v3 sequencing kit.
Data source location	INRAE, UMR PEGASE, Saint-Gilles, France
Data accessibility	Raw RNA-seq data were deposited to the NCBI sequence read archive (SRA) under the accession number SRP144764, https://www.ncbi.nlm.nih.gov/sra/SRP144764. The data can also be accessed through Data INRAE, https://doi.org/10.15454/JJZ3QQ.

**Value of the Data** 

• These data represent hepatic transcriptomes from 4 different duck genetic types (“pure” species and hybrids) fed *ad libitum* or overfed and can be used to analyze responses to overfeeding and differences between genetic types.
• Any researchers involved in liver gene expression and metabolism can benefit from these data and process raw FASTQ files.
• These data can be included in meta-analyses to characterize responses to feeding in different duck breeds.

## 1 Data Description

**Fig. 1:**
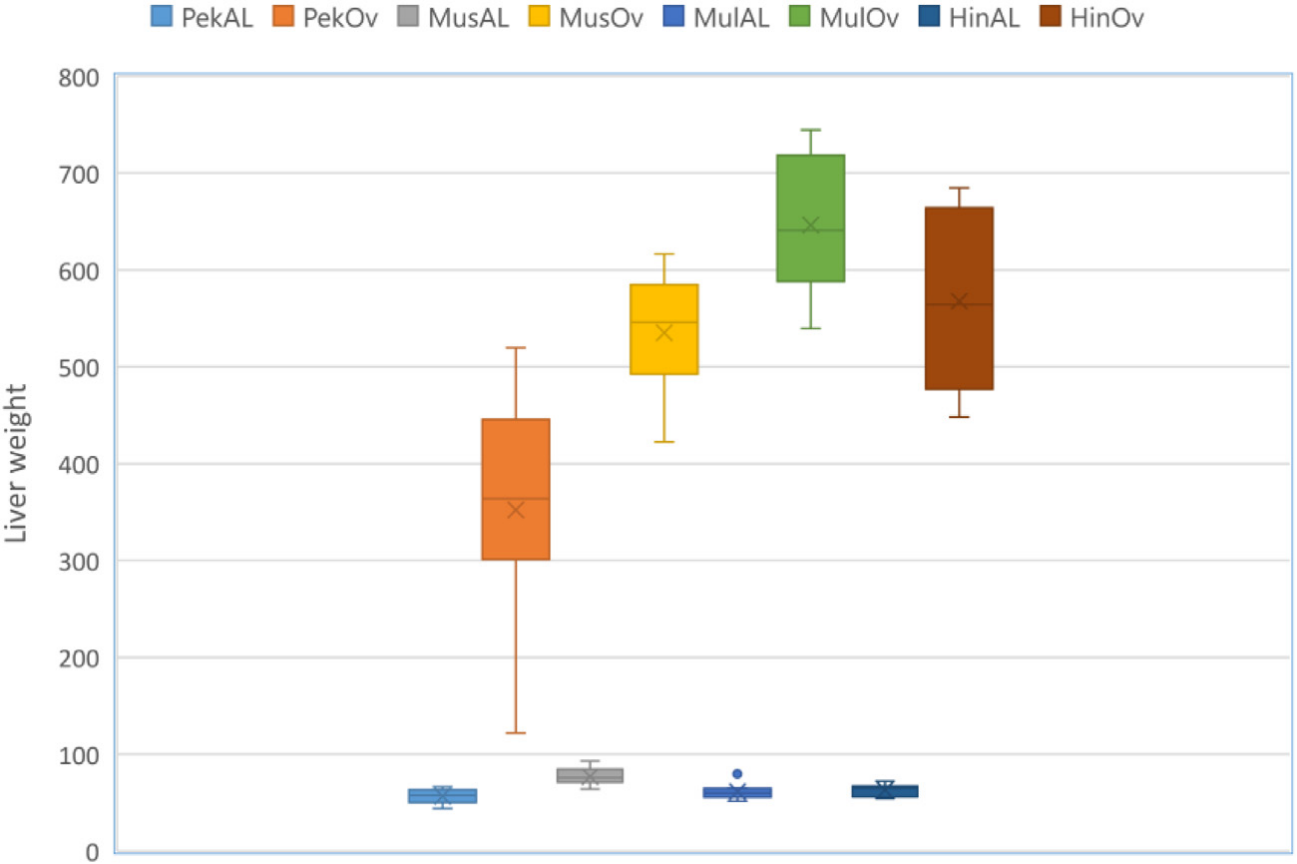
Liver weights (in grams) of Pekin (Pek), Muscovy (Mus), Mule (Mul) and Hinny (Hin) ducks fed *ad libitum* (AL) or overfed (Ov) 14 days.

Data provided in this article were obtained from liver samples of male Pekin ducks fed *ad libitum* (*n* = 10) or overfed (*n* = 10), Muscovy ducks fed *ad libitum* (*n* = 9) or overfed (*n* = 10), Mule duck hybrids fed *ad libitum* (*n* = 10) or overfed (*n* = 10) and Hinny duck hybrids fed *ad libitum* (*n* = 10) or overfed (*n* = 10). A liver weight increase was observed in overfed ducks when compared to ducks fed *ad libitum* ad libitum. This increase was more or less significant depending on the genetic type (Fig. 1).

RNA were extracted from the liver of these ducks and sequenced. Raw sequences FASTQ files were deposited in the NCBI sequence read archive under the study accession number SRP144764 and can also be accessed through Data INRAE with doi:10.15454/JJZ3QQ. Duck sample names, genetic types, feeding conditions and liver weights, SRA accession number (experiment) for direct access to FASTQ files and number of reads are indicated in Table 1.

**Table 1:** Duck liver samples, RNA sequencing and number of reads.

Sample	Genetic type	Feeding	Liver weight (g)	Experiment	Number of reads
PekAL81	Pekin	Ad libitum	58	SRX4048434	54 511 592
PekAL85	Pekin	Ad libitum	60	SRX4048435	63 672 484
PekAL89	Pekin	Ad libitum	63	SRX4048436	54 632 570
PekAL93	Pekin	Ad libitum	64	SRX4048437	100 722 690
PekAL97	Pekin	Ad libitum	67	SRX4048430	62 509 638
PekAL101	Pekin	Ad libitum	44	SRX4048431	29 773 930
PekAL105	Pekin	Ad libitum	47	SRX4048432	65 912 956
PekAL109	Pekin	Ad libitum	51	SRX4048433	64 942 634
PekAL113	Pekin	Ad libitum	57	SRX4048442	41 516 114
PekAL117	Pekin	Ad libitum	57	SRX4048443	40 973 316
PekOv1	Pekin	Overfed	122	SRX4048413	49 425 436
PekOv5	Pekin	Overfed	455	SRX4048414	63 881 908
PekOv9	Pekin	Overfed	519	SRX4048411	58 273 928
PekOv13	Pekin	Overfed	189	SRX4048412	73 132 064
PekOv17	Pekin	Overfed	338	SRX4048409	42 987 014
PekOv21	Pekin	Overfed	343	SRX4048410	39 875 958
PekOv25	Pekin	Overfed	362	SRX4048407	40 240 106
PekOv29	Pekin	Overfed	366	SRX4048408	60 005 478
PekOv33	Pekin	Overfed	385	SRX4048405	62 297 874
PekOv37	Pekin	Overfed	442	SRX4048417	40 498 382
MusAL84	Muscovy	Ad libitum	64	SRX4048444	57 399 836
MusAL88	Muscovy	Ad libitum	72	SRX4048445	42 570 376
MusAL92	Muscovy	Ad libitum	72	SRX4048374	44 882 126
MusAL96	Muscovy	Ad libitum	76	SRX4048375	59 719 512
MusAL104	Muscovy	Ad libitum	70	SRX4048440	52 982 008
MusAL108	Muscovy	Ad libitum	93	SRX4048441	43 990 130
MusAL112	Muscovy	Ad libitum	78	SRX4048438	46 767 682
MusAL116	Muscovy	Ad libitum	91	SRX4048439	53 249 208
MusAL120	Muscovy	Ad libitum	76	SRX4048418	47 410 634
MusOv4	Muscovy	Overfed	472	SRX4048401	59 203 318
MusOv8	Muscovy	Overfed	580	SRX4048400	90 014 240
MusOv12	Muscovy	Overfed	617	SRX4048403	58 607 268
MusOv16	Muscovy	Overfed	422	SRX4048402	62 651 190
MusOv20	Muscovy	Overfed	499	SRX4048397	61 531 550
MusOv24	Muscovy	Overfed	551	SRX4048396	52 439 560
MusOv28	Muscovy	Overfed	513	SRX4048399	40 255 992
MusOv32	Muscovy	Overfed	599	SRX4048398	45 563 618
MusOv36	Muscovy	Overfed	540	SRX4048393	53 283 106
MusOv40	Muscovy	Overfed	555	SRX4048392	54 695 162
MulAL82	Mule	Ad libitum	58	SRX4048447	45 302 210
MulAL86	Mule	Ad libitum	56	SRX4048450	60 094 290
MulAL90	Mule	Ad libitum	61	SRX4048421	71 861 666
MulAL94	Mule	Ad libitum	58	SRX4048415	103 361 470
MulAL98	Mule	Ad libitum	56	SRX4048452	71 673 082
MulAL102	Mule	Ad libitum	65	SRX4048377	40 143 340
MulAL106	Mule	Ad libitum	80	SRX4048404	41 516 888
MulAL110	Mule	Ad libitum	65	SRX4048448	67 585 992
MulAL114	Mule	Ad libitum	63	SRX4048406	72 651 700
MulAL118	Mule	Ad libitum	52	SRX4048451	53 994 728
MulOv2	Mule	Overfed	654	SRX4048420	54 458 584
MulOv6	Mule	Overfed	630	SRX4048389	65 457 472
MulOv10	Mule	Overfed	639	SRX4048388	49 860 540
MulOv14	Mule	Overfed	594	SRX4048391	79 299 348
MulOv18	Mule	Overfed	713	SRX4048390	86 272 820
MulOv22	Mule	Overfed	733	SRX4048376	37 388 720
MulOv26	Mule	Overfed	745	SRX4048416	66 317 938
MulOv30	Mule	Overfed	643	SRX4048395	42 214 570
MulOv34	Mule	Overfed	539	SRX4048394	45 869 644
MulOv38	Mule	Overfed	572	SRX4048449	49 504 406
HinAL83	Hinny	Ad libitum	56	SRX4048378	51 541 602
HinAL87	Hinny	Ad libitum	66	SRX4048422	47 733 202
HinAL91	Hinny	Ad libitum	64	SRX4048423	56 643 460
HinAL95	Hinny	Ad libitum	66	SRX4048424	68 522 628
HinAL99	Hinny	Ad libitum	56	SRX4048425	113 403 456
HinAL103	Hinny	Ad libitum	70	SRX4048426	49 665 684
HinAL107	Hinny	Ad libitum	61	SRX4048427	35 245 860
HinAL111	Hinny	Ad libitum	54	SRX4048428	52 782 990
HinAL115	Hinny	Ad libitum	72	SRX4048429	54 805 168
HinAL119	Hinny	Ad libitum	66	SRX4048419	25 181 580
HinOv3	Hinny	Overfed	485	SRX4048446	49 610 528
HinOv7	Hinny	Overfed	448	SRX4048387	62 190 258
HinOv11	Hinny	Overfed	605	SRX4048386	48 695 180
HinOv15	Hinny	Overfed	524	SRX4048385	66 492 198
HinOv19	Hinny	Overfed	512	SRX4048384	64 544 886
HinOv23	Hinny	Overfed	661	SRX4048383	48 006 454
HinOv27	Hinny	Overfed	674	SRX4048382	49 328 648
HinOv31	Hinny	Overfed	632	SRX4048381	52 348 696
HinOv35	Hinny	Overfed	452	SRX4048380	34 557 162
HinOv39	Hinny	Overfed	685	SRX4048379	57 900 214

## 2 Experimental Design, Materials and Methods

### 2.1 Animals and experimental design

As described previously [[1], [2], [3]] male ducks from four different genetic types, i.e. Pekin, Muscovy and their crossbreed mule (male Muscovy duck x female Pekin duck) and Hinny (male Pekin duck × female Muscovy duck) ducks, were reared under usual conditions of light and temperature at the Experimental Station for Waterfowl Breeding (Unité Expérimentale des Palmipèdes à Foie Gras, INRA Artiguères, France). From hatching to 4 weeks of age, ducks were fed with the starting diet *ad libitum (*free access to the diet). From 4 to 6 weeks of age, they were fed *ad libitum* with the growing diet. From 6 to 12 weeks of age, they were fed with the growing diet at restricted levels appropriate for each genetic type (ranging from 200 to 250 g per duck at the beginning to 360–380 g at the end of the period). At 12 weeks of age, ducks were either fed *ad libitum* with the growing diet or overfed 14 days with high carbohydrate overfeeding diet containing corn and corn meal, respectively indicated as ‘Ad libitum’ and ‘Overfed’ in Table 1. Main characteristics of starting, growing and overfeeding diets are shown in Table 2.

**Table 2:** Characteristics of feeding diets.

Characteristics	Starting (0–4 weeks)	Growing (4–12 weeks)	Overfeeding (12–14 weeks)
ME (kcal/kg)	2830	2850	3330
CP (%)	18.21	15.98	8.28
Lipids (%)	3.34	2.84	3.38
SFA (%)	17.17	6.10	14.52
MUFA (%)	24.98	28.36	27.44
PUFA (%)	57.85	55.54	58.02

ME: metabolisable energy; CP: crude protein; SFA, MUFA, PUFA: saturated, mono-unsaturated and poly-unsaturated fatty acids. Feed for overfeeding contained corn (25%), corn meal (35%) and water (40%).

**Table 3:** Multiplexing per lane (L) of duck liver RNA libraries and tagging before sequencing.

	Tag1	Tag2	Tag3	Tag4	Tag5	Tag6
	ATCACG	TTAGGC	ACTTGA	GATCAG	TAGCTT	GGCTAC
L1	PekOv1	MusAL88	HinOv11	MulAL94	MulOv18	PekAL101
L2	MulAL82	PekOv5	MusAL92	HinOv15	HinAL99	MulOv22
L3	HinOv3	MulAL86	PekOv9	MusAL96	MusOv20	HinAL103
L4	MusAL84	HinOv7	MulAL90	PekOv13	PekAL97	MusOv24
L5	MulOv2	PekAL85	MusOv12	HinAL95	HinOv19	MulAL102
L6	PekAL81	MusOv8	HinAL91	MulOv14	MulAL98	PekOv21
L7	MusOv4	HinAL87	MulOv10	PekAL93	PekOv17	MusAL104
L8	HinAL83	MulOv6	PekAL89	MusOv16		HinOv23
L9	PekOv25	HinAL107	MulOv30	MusAL112	HinOv35	PekAL113
L10	MulAL106	MusOv28	PekAL109	HinOv31	MusAL116	MulOv34
L11	HinOv27	PekAL105	MusOv32	MulAL110	PekOv33	HinAL115
L12	MusAL108	MulOv26	HinAL111	PekOv29	MulAL114	MusOv36
L13	PekOv37	MulAL118	HinOv39	MusAL120	MulOv38	PekAL117
L14	HinAL119	MusOv40				

Hexamer tag sequences are indicated under tag numbers.

Fourteen hours after the last meal, ducks were rendered unconscious and unable to feel pain by electronarcosis and were slaughtered by neck sectioning and bleeding. Immediately after bleeding, liver were weighted (Table 1), and samples were collected, rapidly frozen in liquid nitrogen and stored at − 80 °C until RNA extraction.

### 2.2 RNA preparation and sequencing

Total RNA were extracted from liver samples using NucleoSpin® RNA L kit (Macherey-Nagel SARL, Hoerdt, France) including guanidinium thiocyanate, silica membrane and on-column RNase-free DNase digestion according to the manufacturer's instructions without modification. RNA concentration was determined using a NanoDrop ND-1000 Spectrophotometer (Thermo Scientific, Illkirch, France). Quality and integrity of RNA were checked using Lab-on-a-Chip Eukaryote Total RNA Nano chip and Bionalyzer 2100 device (Agilent Technologies France, Massy, France). RNA with absorbance ratio λ260 nm/λ280 nm and λ260 nm/λ230 nm >1.8 and RNA integrity number (RIN) > 7.4 were selected (resulting in 9–10 RNA samples per genetic type and per diet).

Libraries preparation and sequencing experiments were performed at the Genotoul genomics facility GeT-PlaGe (http://get.genotoul.fr/en/). RNA libraries were prepared according to Illumina's protocols without modification using the Illumina TruSeq RNA Sample Prep Kit v2 (Illumina, San Diego, CA). PolyA+ mRNA were first isolated using oligo(dT) beads. Then, mRNA were fragmented and reverse transcribed into double stranded cDNA. Adapters and hexamer tags (Table 3) were ligated for subsequent identification. Ten cycles of PCR were applied to amplify libraries. Library quality was assessed using an Agilent Bioanalyser (Agilent Technologies France, Massy, France) and libraries were quantified by qPCR using the Kapa Library Quantification Kit. RNA sequencing was performed with the Illumina HiSeq2000 SBS v3 sequencing kit on HiSeq2000 Illumina platform using a paired-end read length of 2 × 100 pb. The libraries were sequenced on 14 different lanes, 6 samples per lane randomly selected as indicated in Table 3. Numbers of raw reads are shown in Table 1 with an average 56.1 ± 1.6 M of reads per sample.

The reads were of good quality (quality scores above 28) as controlled using FastQC (http://www.bioinformatics.babraham.ac.uk/projects/fastqc/).

## Ethics Statement

Liver samples were collected in a previous study [3]. They were reused later for RNA extraction and sequencing as described in this data paper, thus complying with the “reduce” recommendation of the 3R rules [4]. The animal experiments (number C22 237) were performed in accordance with the EU Directive 2010/63/EU for animal experiments. Animal experiments also complied with the ARRIVE guidelines [5].

## CRediT Author Statement

**Frédéric Hérault:** Conceptualization, Methodology, Formal analysis, Resources. **Elisabeth Baéza:** Conceptualization, Resources. **Christian Diot:** Conceptualization, Methodology, Supervision, Project administration, Writing- Original draft and Funding acquisition.

## Declaration of Competing Interest

The authors declare that they have no known competing financial interests or personal relationships which have or could be perceived to have influenced the work reported in this article.
